# Association of Maternal Weight and Gestational Weight Gain with Maternal and Neonate Outcomes: A Prospective Cohort Study

**DOI:** 10.3390/jcm8122074

**Published:** 2019-11-27

**Authors:** Damien Bouvier, Jean-Claude Forest, Emilie Dion-Buteau, Nathalie Bernard, Emmanuel Bujold, Bruno Pereira, Yves Giguère

**Affiliations:** 1 Biochemistry and Molecular Genetic Department, CHU Clermont-Ferrand, Université Clermont-Auvergne, Faculty of Medicine, CNRS 6293, INSERM 1103, GReD, 63000 Clermont-Ferrand, France; dbouvier@chu-clermontferrand.fr; 2 Centre de Recherche du CHU de Québec-Université Laval, Department of Molecular Biology, Medical Biochemistry and Pathology, Faculty of Medicine, Québec City, G1V 0A6, Canada; jean-claude.forest@chudequebec.ca; 3 Centre de Recherche du CHU de Québec-Université Laval, Québec City, G1L 3L5, Canada; emilie.dion-buteau.1@ulaval.ca (E.D.-B.); nathalie.bernard@crchudequebec.ulaval.ca (N.B.); 4 Department of Obstetrics, Gynecology and Reproduction, Faculty of Medicine, Université Laval, Québec City, G1V 0A6, Canada; Emmanuel.bujold@crchudequebec.ulaval.ca; 5 Biostatistics Unit (DRCI), CHU de Clermont-Ferrand, 63000 Clermont-Ferrand, France; bpereira@chu-clermontferrand.fr

**Keywords:** gestational weight gain, IOM recommendations, gestational diabetes mellitus, hypertensive disorders of pregnancy, caesarean delivery, macrosomia, small for gestational age, large for gestational age, neonatal hypoglycemia, group-based multi-trajectory modelling

## Abstract

We investigated the association of outcomes with pre-pregnancy body mass index (ppBMI), Institute of Medicine (IOM) recommendations about gestational weight gain, and weight gain trajectories during pregnancy. A prospective cohort of 7866 pregnant women was recruited. ppBMI and weight gain at each follow up visit were collected. The outcomes were gestational diabetes mellitus (GDM), hypertensive disorders of pregnancy (HDP), caesarean delivery, macrosomia, small (SGA) and large (LGA) for gestational age, neonatal hypoglycemia. Group-based multi-trajectory modelling was used for weight kinetics during pregnancy. In the third trimester, 53.8% of women were above IOM recommendations, with an increased relative risk (RR) of HDP (1.91 (1.40–2.61)), caesarean (1.34 (1.15–1.56)), macrosomia (2.17 (1.77–2.67)), LGA (2.26 (1.83–2.80)), and hypoglycemia (1.89 (1.12–3.18)). Women with a weight gain above IOM recommendations in the second trimester who normalized their weight gain in third trimester had, compared to those who remained above IOM recommendations, fewer events of HDP (2.8% versus 5.3%, *p* = 0.008), caesarean delivery (16.9% versus 22%, *p* = 0.006), macrosomia (8.3% versus 14.2%, *p* < 0.001), and LGA (7% versus 13.2%, *p* < 0.001). Multi-trajectory modelling identified three profiles with continued variation in RR of complications, including GDM. Weight gain above IOM recommendations increased the risk of perinatal complications. A correction of excessive weight gain in the second trimester reduces these risks.

## 1. Introduction

The prevalence of obesity has been increasing significantly in many countries in recent years [1,2]. For example, in the United States in 2008, 58.5% of reproductive age women are overweight or obese (body mass index ≥25 kg/m^2^) [3,4] and the numbers are still increasing [1,2]. Pre-pregnancy body mass index (ppBMI), excessive and insufficient weight gain have been associated with adverse pregnancy outcomes, including small for gestational age (SGA), large for gestational age (LGA), macrosomia, caesarean delivery, gestational diabetes mellitus (GDM), preeclampsia, postpartum weight retention, and offspring obesity [5,6,7,8]. In 2009, the Institute of Medicine (IOM) updated its recommendations on gestational weight gain according to ppBMI [9]. These guidelines were developed to minimize the negative health consequences for both mother and fetus of inadequate or excessive gain. They incorporated World Health Organization (WHO) categories of ppBMI maternal body mass index (BMI calculated as weight in kilograms divided by height in squared meters; BMI for underweight, <18.5; normal weight, 18.5–24.9; overweight, 25–29.9; and obese, ≥30) [10], and recommended less gestational weight gain for obese women. A meta-analysis of over one million pregnant women highlighted that 47% had gestational weight gain greater than IOM recommendations, while 23% had gestational weight gain less than IOM recommendations, leaving only 30% of women considered with adequate gestational weight gain. Gestational weight gain above recommendations was associated with higher risk of LGA, macrosomia, and caesarean delivery [11]. A retrospective study of chart abstractors from 29,861 women in 25 American hospitals found similar results with the addition of an association between gestational weight gain above the IOM recommendations and shoulder dystocia and neonatal hypoglycemia [12].

In this context, we revisited the association of maternal pre-pregnancy weight and gestational weight gain with maternal and neonatal outcomes, taking advantage of a large prospective cohort of 7866 pregnant women recruited at their first prenatal visit to the perinatal clinic of the University Hospital in Quebec City, Canada. This clinic was the entry point for health care services for all pregnant women of the region. The important collection of data during the constitution of this cohort from an unselected low-risk population made it possible to evaluate IOM recommendations in the second and third trimesters of pregnancy, and to study the kinetics of weight gain using group-based trajectory modelling. This trajectory approach allows for an original study of the evolution of women’s weights during their pregnancies independent of the categories or recommendations of WHO or IOM.

## 2. Experimental Section

### 2.1. Study Design and Participants

**Figure 1:**
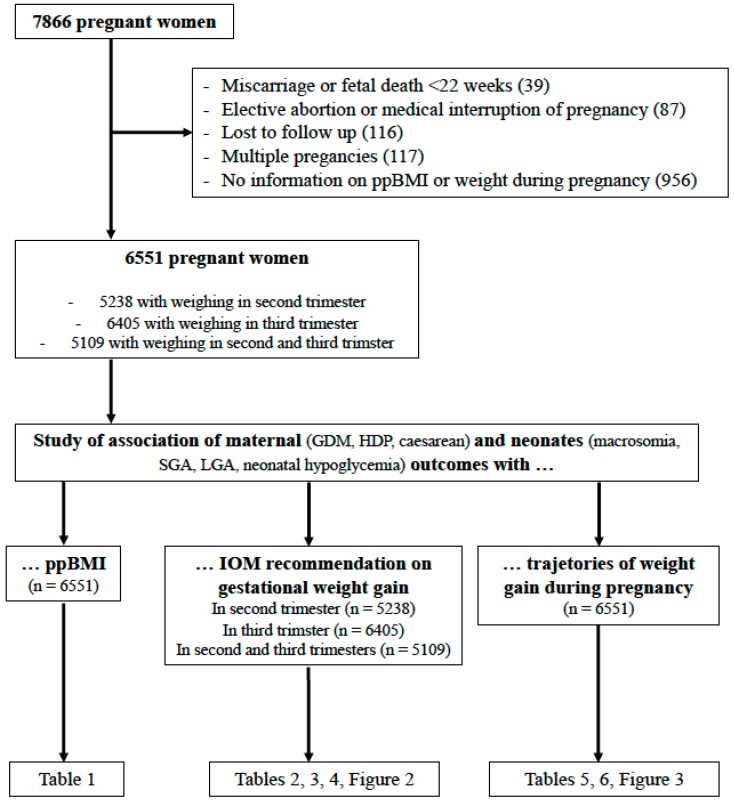
Flow chart of the study IOM: Institute of Medicine; GDM: gestational diabetes mellitus; HDP: hypertensive disorders of pregnancy; LGA: large for gestational age; ppBMI: pre-pregnancy body mass index; SGA: small for gestational age.

This study is based on a large prospective cohort of 7866 pregnant women recruited at the “CHU de Québec-Université Laval” from April 2005 to March 2010 at their first prenatal visit to the perinatal clinic of the institution (A comprehensive Healthy Pregnancy Initiative from the Institute for Human Development, Child and Youth Health, Canadian Institutes of Health Research). This cohort has been described in detail elsewhere [13,14,15,16,17]. Pregnant women aged 18 years or older without chronic hepatic or renal disease were eligible to participate in the study. Participants gave written informed consent and the study was approved by the “CHU de Québec” Ethics Review Board (initial approval date: 9 November 2004, Project 5-04-10-01 [95.05.17l SC12-01-159). Data have been collected prospectively about socioeconomic characteristics, lifestyle, medical, history of mothers, events at delivery, and complications in neonates. Pre-pregnancy weight was collected. During the medical visit of the first trimester, pregnant women were measured for height and weighed. During the medical visits of the second and third trimesters, pregnant women were weighed. Exclusion criteria for this study were pregnant women without information on ppBMI or weight during pregnancy, miscarriage or fetal death before 22 weeks of gestation, pregnancy termination (elective abortion or medical interruption of pregnancy), multiple pregnancies, and lost to follow up (Figure 1). 

Women were classified by their ppBMI according to WHO categories (BMI calculated as weight in kilograms divided by height in meters squared; BMI for underweight, <18.5; normal weight, 18.5–24.9; overweight, 25–29.9; obese, ≥30) [10]. Knowing the ppBMI, the weight gain in the first trimester and the gestational age at the time of the weighing of the second and third trimesters, we estimated in the second (T2) and the third trimester (T3) whether women’s weight gain was within IOM guidelines at the end of pregnancy (total recommended weight gain: 12.5–18 kg for BMI <18.5, 11.5–16 kg for BMI 18.5–24.9, 7–11.5 kg for BMI 25–29.9, 5–9 kg for BMI ≥30). Weight gain values within IOM guidelines were classified as T2N (N for Normal) in the second trimester and T3N in the third trimester of pregnancy. Weight gain values below IOM guidelines were classified as T2L (L for Low) in the second trimester and T3L in the third trimester of pregnancy. Weight gain values above IOM guidelines were classified as T2H (H for High) in the second trimester and T3H in the third trimester of pregnancy.

### 2.2. Studied Criteria

Maternal outcomes studied in the different categories were gestational diabetes mellitus (GDM), hypertensive disorders of pregnancy (HDP), and mode of delivery (caesarean or not). GDM diagnosis was established according to the Canadian Diabetes Association 2013 Clinical Practice Guidelines [18]. According to these recommendations, most women (90.7%) had a 50 g glucose challenge test (GCT) between 24 and 28 weeks of gestation, followed by a 75 g oral glucose tolerance test (OGTT) if the result of the GCT was between 7.8 and 10.2 mmol/L. GDM was diagnosed if the result of the GCT was ≥ 10.3 mmol/L or if one or more values equaled or exceeded the thresholds of 5.3, 10.6 and 9 mmol/L at 0, 1 and 2 h, respectively, during the OGTT. Diagnosis of HDP was made by a senior obstetrician according to the classification of the Society of Obstetricians and Gynaecologists of Canada based on information retrieved from medical records and includes gestational hypertension (GH) and preeclampsia. GH was defined as de novo hypertension (systolic blood pressure ≥140 mmHg and/or diastolic blood pressure ≥90 mmHg) after 20 weeks of pregnancy. Preeclampsia was defined as GH with proteinuria (≥300 mg in a 24-h urine collection or ≥2+ on dipstick in a random sample) or pre-existing hypertension and new or worsening proteinuria. Neonatal outcomes studied were macrosomia (defined as a birth weight above 4000 g), small for gestational age (SGA indicated by birth weight less than the 10th percentile for gestational age), large for gestational age (LGA indicated by birth weight greater than the 90th percentile for gestational age), and neonatal hypoglycemia requiring treatment as recommended by the Canadian Pediatric Society [19].

We also studied two socioeconomic characteristics: mother’s level of education (no diploma versus diploma (high school or college or university diploma)) and familial annual income (> or < 15,500 Canadian dollars).

### 2.3. Statistical Analysis

All analyses were performed using Stata software (Version 13, StataCorp, College Station, TX) and R software [20] for a two-sided Type I error of 5%. Patient’s characteristics were expressed as mean ± standard-deviation (SD) or median (interquartile range, minimum and maximum) for continuous data (assumption of normality assessed by using the Shapiro-Wilk test) and as numbers and associated percentages for categorical parameters. 

To study the association of maternal and neonatal outcomes with ppBMI and IOM recommendations on gestational weight gain, univariate and multivariable analyses were performed using robust (standard-errors) Poisson generalized-linear-model regression (package gllamm). Results were expressed as relative-risk ratios (RR) and 95% confidence intervals (95% CI). The covariates were determined according to univariate results and clinical relevance. More precisely, for all outcomes except GDM, the adjustment of RR was based on the age of mothers, parity, smoking during pregnancy, hypertension before pregnancy, diploma of mothers, familial annual incomes and diabetes before pregnancy. For GDM, the adjustment of RR was based on the age of mothers, parity, smoking during pregnancy, hypertension before pregnancy, diploma of mothers, and familial annual incomes. Attention has been paid to the study of multicollinearity and interactions between covariates: (1) studying the relationships between the covariables and (2) evaluating the impact to add or delete variables on multivariable model. 

To identify distinctive trajectories of weight evolution, a semi-parametric mixture model (group-based trajectory model-GBTM) was performed to characterize the relationship between weight and time for each trajectory, the shape of the trajectory and the estimated proportion of the population belonging to each trajectory [21], involving an approach which gathers individuals into meaningful subgroups that show statistically similar trajectories, i.e., identify groups of distinctive trajectories which are summarized by a finite set of different polynomial functions of time [22,23]. Rather than assuming a priori, i.e., the existence of trajectories of a specific form, the method allows the trajectories to emerge from the data. This model allows for data grouping using different parameter values for each group distribution. Groupings may identify distinct subpopulations. Maximum likelihood is used for the estimation of the model parameters. The maximization is performed using a general quasi-Newton procedure. The fundamental concept of GBTM is the distribution of weight conditional on time. That is, the distribution of weight trajectories denoted by *P(Y_i_|Time_i_)* where the random vector *Y_i_* represents individual i’s longitudinal sequence of weight and the vector *Time_i_* represents individual i’s time when each of those measurements is recorded. The group-based trajectory model assumes that the population distribution of trajectories arises from a finite mixture of unknown order J. The analysis provides a formal way to determine the best-fit number of trajectories and a precision estimate of group membership allocation which can be expressed using observed probabilities and posteriori probabilities. These values were expected as close as possible. The posterior probabilities of group membership measure the likelihood for each patient to belong to its assigned group. Nagin recommends that the average posterior probabilities should exceed a minimum of 0.70 for each group [24]. Furthermore, the best-fitting model was selected according to the Bayesian information criterion (BIC). Then, the continuous variables were compared between independent groups (trajectories) by ANOVA, or Kruskal–Wallis test if the assumptions of ANOVA were not met. The homoscedasticity was analyzed using the Bartlett test. When appropriate, post-hoc tests were performed taking into account multiple comparisons (Tukey–Kramer post ANOVA and Dunn after Kruskal–Wallis). The comparisons between independent trajectories were carried out using Chi-squared or Fischer’s exact tests for categorical variables. When appropriate, a post-hoc test was performed (Marascuillo procedure).

The relationships between these trajectories of weight and perinatal outcomes were studied as aforementioned.

## 3. Results

### 3.1. Description of the Cohort

Of the 6551 women fulfilling the inclusion criteria (Figure 1), 344 (5.3%) were underweight, 4045 (61.7%) presented a normal ppBMI, 1351 (20.6%) were overweight, and 811 (12.4%) were obese (Table 1). The mean age at recruitment was 30 years (SD: 4.3) and underweight women were significantly younger (29.1 years; mean (SD: 4.3)) (Table 1). Over 98% of study subjects were Caucasians. 

### 3.2. Association of Maternal and Neonatal Outcomes with ppBMI

The results are presented in Table 1. Overweight and obese women were at increased relative risk of GDM, HDP, caesarean delivery and giving birth to a macrosomic or LGA neonate. Obese women showed a decreased relative risk to deliver an SGA neonate. Underweight women showed an increased relative risk of giving birth to an SGA neonate and have a significantly lower level of education and annual family income. From underweight to obese women, the proportion of GDM, HDP, caesarean, macrosomia, LGA and SGA monotonically varied. 

### 3.3. Association of Maternal and Neonatal Outcomes with IOM Recommendations on Gestationnal Weight Gain

Results from second trimester of pregnancy are presented in Table 2. In the second trimester, 14.4% of women were below IOM recommendations for gestational weight gain and 56.5% were above IOM recommendations. Compared to pregnant women who followed the IOM recommendations, women with a gestational weight gain above IOM recommendations had an increased relative risk of HDP, caesarean, delivering a macrosomic or an LGA neonate, and of having a baby with neonatal hypoglycemia. Women with gestational weight gain below IOM recommendations had an increased relative risk of GDM. 

Results from third trimester of pregnancy are presented in Table 3. In the third trimester, 13.3% of women were below IOM recommendations and 53.8% were above IOM recommendations. Women with a gestational weight gain above IOM recommendations showed an increased relative risk of HDP, caesarean delivery, giving birth to a macrosomic or an LGA neonate, and neonatal hypoglycemia. They presented a decreased relative risk of giving birth to an SGA neonate. Women with a gestational weight gain below IOM recommendations were at increased relative risk of GDM in the second trimester and delivering an SGA neonate.

In both second and third trimester, women outside the IOM recommendations have a significantly lower level of education.

**Figure 2:**
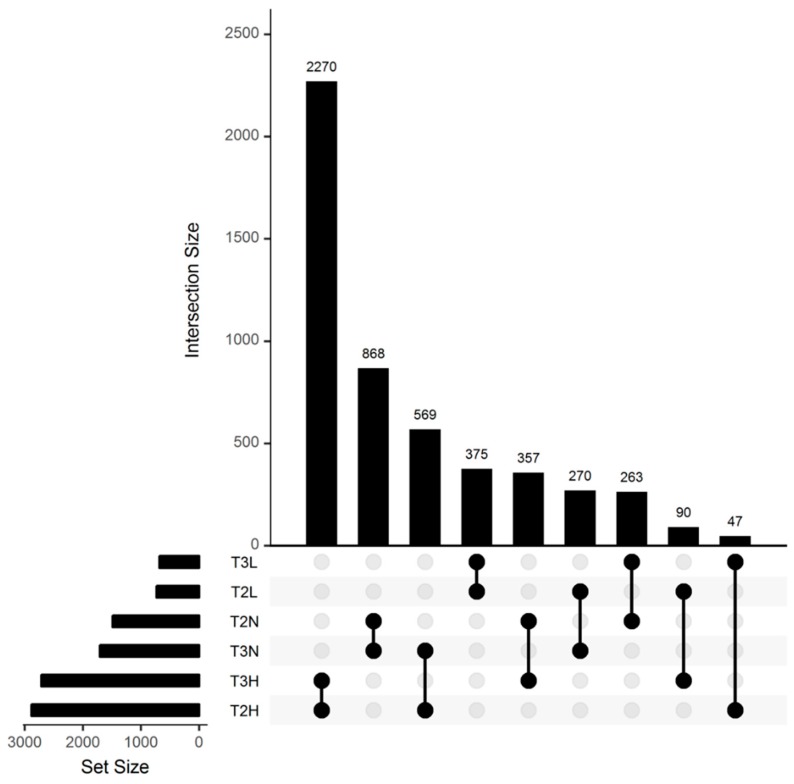
Evolution of gestational weight gain according to IOM recommendations between the second and third trimesters of pregnancy.

Of 5109 women with weighing data in the second and third trimesters of pregnancy, 2270 (44.4%) were above IOM recommendations in both the second and third trimester (T2H and T3H) and 616 (12%) were above IOM recommendations in the second trimester but within (569) or below (47) recommendations in the third trimester (T2H and T3N or T3L) (Figure 2). Women with a gestational weight gain above IOM recommendations in the second trimester of pregnancy but within or below IOM recommendations in the third trimester (T2H and T3N or T3L) had fewer events of HDP, caesarean delivery, macrosomia or LGA than women who remained above IOM recommendations in the third trimester (T2H and T3H) (Table 4A). Women with a gestational weight gain within IOM recommendations in the second trimester of pregnancy but above IOM recommendations in the third trimester (T2N and T3H) had more events of HDP, macrosomia or LGA, neonatal hypoglycemia than women who remained within or below IOM recommendations in the third trimester (T2N and T3N or T3L) (Table 4B). 

### 3.4. Trajectories of Weight Gain during Pregnancy

**Figure 3:**
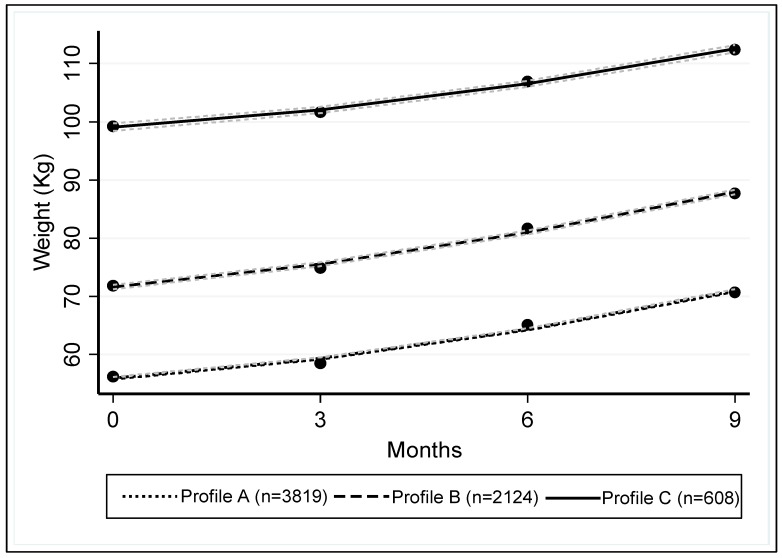
Trajectories of weight gain during pregnancy. Each trajectory is framed by 95% confidence intervals. The estimated slope for profile A was 2.34 (95%CI: 2.25; 2.45), 3.01 (95%CI: 2.82; 3.19) for profile B and 2.47 (95%CI: 2.01; 2.93) for profile C. The slope for profile C is significantly different than the slopes for profiles A (*p* < 0.001) and B (*p* = 0.01). CI: confidence interval.

Group-based multi-trajectory modelling identified three profiles (A, B and C) of weight gain kinetics during pregnancy (Figure 3). The characteristics of these profiles are presented in Table 5. 

The average a posteriori probability of being in profile A was 97.1%. For those in profile B, the average probability was 95.2%, and for those in profile C, the average probability was 97.5%. Furthermore, for profile A, the observed probabilities of groups versus the probability based on the posteriori probabilities were 58.3% and 58.0%; for profile B, 32.4% and 32.8%, respectively; and for profile C, 9.3% and 9.2%.

The distribution of ppBMI categories was significantly different between the three profiles (*p* <0.001). Among the 3819 (58.3%) women in profile A, 85.6% had a normal ppBMI, while 50.9% of the 2124 (32.4%) women in profile B were overweight, and 89.3% (53% with BMI >35 kg·m^−2^) of the 608 (9.3%) women in profile C were obese. Women of profile B gained significantly more weight in the three trimesters of pregnancy than the other two profiles (*p* = 0.001).

From profiles A to C, the proportion of GDM, HDP, caesarean, macrosomia, LGA neonates and SGA neonates monotonically varied (Table 6). Women of profiles B and C were at increased relative risk of GDM, HDP, caesarean, and delivering a macrosomic or LGA neonate. They presented a decreased relative risk of giving birth to an SGA neonate. Women of profile C presented an increased relative risk of neonatal hypoglycemia (Table 6). 

## 4. Discussion

We studied the association of seven maternal and neonatal outcomes with ppBMI, IOM recommendations on gestational weight gain and weight gain trajectories during pregnancy. To our knowledge, we showed for the first time with an independent cohort, the relationship between IOM recommendations for gestational weight gain in the second trimester and perinatal outcomes. Furthermore, we showed that compliance with recommendations through both the second and third trimesters reduced the proportion of HDP, caesarean delivery, macrosomia, and LGA. Inversely, non-compliance with the recommendations between the second and third trimesters (i.e., going from within to above recommendations) increased the proportions of HDP, macrosomia and LGA. Finally, using group-based multi-trajectory modelling, we identified three profiles of pregnancy weight gain kinetics: profile A of women with normal ppBMI, profile B of rather overweight women with increased weight gain during pregnancy compared to the other two profiles and profile C of obese women. From profile A to profile C, the relative risk of GDM, HDP, caesarean delivery, macrosomia and LGA increased.

First, we validated our cohort by confirming that overweight and obese women show an increased relative risk of GDM, HDP, caesarean delivery and delivering a macrosomic or LGA neonate [5,25]. We also confirmed that underweight women are at increased relative risk of giving birth to SGA neonates and at decreased relative risk of caesarean, giving birth to a macrosomic or an LGA neonate [5,25]. Secondly, as demonstrated in two recent studies, including a meta-analysis [11,12], we validated our cohort by highlighting that in the third trimester of pregnancy, 13.3% of women were below IOM recommendations, while 53.8% were above IOM recommendations. Women with a gestational weight gain above IOM recommendations were at increased relative risk of HDP, caesarean, giving birth to a macrosomic or LGA neonate, and neonatal hypoglycemia.

With 53.8% of women above the IOM recommendations for gestational weight gain during pregnancy, it is questionable whether these are not too severe. However, it is noteworthy that those above IOM recommendations in the third trimester had a 1.92-fold increased risk of HDP, a 2.13-fold increased risk of macrosomia, a 2.28-fold increased risk of LGA and a 1.97-fold increased risk of neonatal hypoglycemia. Hyperinsulinism and insulin resistance caused by obesity and excessive weight gain [26] may explain the increase in these risks [27,28,29]. Our findings of the association of maternal and neonatal outcomes with IOM recommendations as early as the second trimester of pregnancy, and that compliance with recommendations between the second and third trimesters reduced this association, may have a significant impact on clinical practice. Indeed, these results should encourage physicians to confront pregnant women’s weight gain in the second trimester with IOM recommendations and to trigger appropriate interventions in cases of non-compliance. This is quite relevant considering that dietary and lifestyle interventions during pregnancy can reduce maternal gestational weight gain and improve outcomes for both mother and baby. Diet-based interventions are the most effective; they result in a reduction of maternal gestational weight gain and improve obstetric outcomes [30]. IOM recommendations are thus relevant. Progress has been made on pregnancy weight gain control in the second trimester, but further efforts should be made in the third trimester. Indeed, we highlighted that 78.6% of women above the IOM recommendations in the second trimester of pregnancy remained above the recommendations in the third trimester. While we validated that obesity increases GDM risk by a factor of 3.9, it may seem surprising to note that GDM was more prevalent among women below IOM recommendations of gestational weight gain compared to those with normal weight gain. However, this has been previously observed [12], and is probably attributed to a bias related to the treatment of women screened as diabetics and its potential effect on gestational weight gain. However, this may not explain the observed risk increase in the second trimester. This would need to be further studied.

Group-based multi-trajectory modelling allowed identifying three profiles of the kinetics of gestational weight gain independently of any recommendations. Using this approach, we identified three subgroups that clearly differed in ppBMI and gestational weight gain with a monotonic variation of the risk of the outcomes under study, including GDM. Obesity and particularly severe obesity (BMI >35 kg·m^−2^) remain major risk factors of maternal and neonatal complications regardless of weight gain [5], while the combination of pre-pregnancy overweight and increased weight gain during pregnancy represents an intermediate risk group. 

An interventional study, triggering a medical intervention in patients with excessive weight gain according to IOM recommendations in the second trimester, would definitively confirm our results and the practical relevance of the IOM recommendations. The proportion of maternal and neonatal outcomes would be compared between an interventional group and a control group.

A strength of our prospective study was the homogeneity of our general population. With predominantly Caucasian (>98%) women and a public health system where all pregnant women have access to similar pregnancy follow up and monitoring, the possibility of sampling bias is reduced. With regards to socioeconomic aspects, the free access to perinatal care for the Quebec population may limit biases and contributes to generalizability of the results. However, the socioeconomic status had an effect on the perinatal outcomes even within a similar setting of universal access to health care [31]. This is why we integrated the mother’s level of education and familial annual income in the adjustments of relative risks. Moreover, the homogenous origin of our cohort does not allow measuring the impact of ethnicity such as African origin. Women of African origin are more susceptible to being affected by obesity and excessive weight gain during pregnancy [32]. Gestational weight gain recommendations specific for the Asian population are also necessary [33]. Thus, external validity of our results should be tested in populations of different ethnic backgrounds. The lack of information on ppBMI or weight during pregnancy for 956 (12%) women of the cohort could be considered as a potential limitation. The exclusion of 956 (12.2%) women from the cohort due to the absence of information on ppBMI or weight during pregnancy may represent a selection bias. As explained above, the fact that some of the results confirm those of previous studies limits this bias [34].

## 5. Conclusions

Women with a gestational weight gain above IOM recommendations from the second trimester of pregnancy were at increased risk of hypertensive disorders of pregnancy, caesarean delivery, and delivering a macrosomic or large-for-gestational-age neonate, and of neonatal hypoglycemia. While ppBMI represents a major determinant of pregnancy outcomes, the correction of excessive gestational weight gain in the second trimester can reduce these risks. These findings constitute a proof of concept of the clinical utility of the IOM recommendations and will encourage physicians to confront pregnant women’s weight gain in the second trimester with IOM recommendations and to trigger intervention when indicated. Pre-pregnancy obesity and particularly severe obesity (BMI >35 kg·m^−2^) remain a high risk of maternal and neonatal complications regardless of weight gain or recommendations. The combination of pre-pregnancy overweight and excessive weight gain during pregnancy represents an intermediate risk group. 

## Figures and Tables

**Table 1:** Association of maternal and neonatal outcomes with pre-pregnancy body mass index.

		Pre-Pregnancy Body Mass Index in kg·m^−2^				Continuity between a, b, c and d
		a: <18.5 (Underweight)	b: 18.5–24.9 (Normal)	c: 25–29.9 (Overweight)	d: >30 (Obese)	
*n* (%)		344 (5.3)	4045 (61.7)	1351 (20.6)	811 (12.4)	/
Mean age of mothers (SD) in years		29.1 * (4.3)	30 (4.3)	30 (4.3)	30.1 (4.2)	/
Mean gestational age at delivery (SD) in months		39.2 ^ (1.5)	39.5 ** (1.4)	39.5 (1.5)	39.3 (1.6)	/
Mothers with no diploma in %		6.7 ***	3.1	4.4 ^^	5 ^^	/
Familial annual income <15,500 Canadian dollars in %		7.9 ***	2.8	2.7	3.1	/
Maternal outcomes	Gestational diabetes mellitus in %	4.6	4.9	8.7	17.5	<0.001
	RR (CI 95%), *p*	0.95 (0.56–1.60), 0.84	1	1.86 (1.47–2.36), <0.001	4.12 (3.27–5.19), <0.001	
	aRR (CI 95%), *p*	0.86 (0.48–1.54), 0.62	1	1.70 (1.32–2.20), <0.001	3.69 (2.87–4.76), <0.001	
	Hypertensive disorders of pregnancy in %	2.9	3.1	5.9	9.6	<0.001
	RR (CI 95%), *p*	0.95 (0.49–1.82), 0.87	1	1.96 (1.47–2.62), <0.001	3.36 (2.51–4.52), <0.001	
	aRR (CI 95%), *p*	0.99 (0.49–1.99), 0.98	1	1.87 (1.37–2.55), <0.001	3.13 (2.25–4.35), <0.001	
	Caesarean delivery in %	11.6	18.3	22.2	28.9	<0.001
	RR (CI 95%), *p*	0.59 (0.42–0.82), 0.002	1	1.27 (1.09–1.48), 0.002	1.81 (1.52–2.14), <0.001	
	aRR (CI 95%), *p*	0.66 (0.46–0.95), 0.025	1	1.29 (1.09–1.52), 0.003	1.85 (1.53–2.23), <0.001	
Neonate outcomes	Macrosomia in %	4.6	8.6	13.6	18	<0.001
	RR (CI 95%), *p*	0.52 (0.31–0.87), 0.013	1	1.68 (1.39–2.03), <0.001	2.33 (1.89–2.88), <0.001	
	aRR (CI 95%), *p*	0.60 (0.35–1.03), 0.063	1	1.62 (1.31–1.99), <0.001	2.32 (1.85–2.93), <0.001	
	Large weight for gestational age in %	3.8	7.6	13.7	18	<0.001
	RR (CI 95%), *p*	0.48 (0.27–0.84), 0.01	1	1.92 (1.58–2.33), <0.001	2.66 (2.15–3.29), <0.001	
	aRR (CI 95%), *p*	0.57 (0.32–1.01), 0.054	1	1.79 (1.45–2.20), <0.001	2.48 (1.96–3.14), <0.001	
	Small weight for gestational age in %	11.6	5.7	5.1	3.7	<0.001
	RR (CI 95%), *p*	2.19 (1.53–3.12), <0.001	1	0.88 (0.67–1.17), 0.39	0.64 (0.44–0.95), 0.026	
	aRR (CI 95%), *p*	1.87 (1.23–2.84), 0.003	1	0.88 (0.65–1.20), 0.41	0.59 (0.38–0.93), 0.023	
	Neonatal hypoglycemia in %	1.5	1.4	1.9	2	0.38
	RR (CI 95%), *p*	1.07 (0.43–2.69), 0.89	1	1.42 (0.89–2.28), 0.14	1.46 (0.83–2.56), 0.19	
	aRR (CI 95%), *p*	1.20 (0.47–3.07), 0.71	1	1.57 (0.95–2.58), 0.08	1.51 (0.82–2.80), 0.19	

aRR: adjusted relative risk; CI: confidence interval; IOM: Institute of Medicine; SD: standard deviation. Adjustment of RR was made for all outcomes on age of mothers, parity, smoking during pregnancy, hypertension before pregnancy, diploma of mothers, familial annual incomes and for all outcomes except gestational diabetes mellitus on diabetes before pregnancy. * Different from b, c and d (*p* = 0.001); ^ Different from b and c (*p* = 0.006); ** Different from d (*p* = 0.006); *** Different from b (*p* = 0.001); ^^ Different from b (*p* < 0.05).

**Table 2:** Association of maternal and neonatal outcomes with IOM recommendations on gestational weight gain in the second trimester of pregnancy.

		Gestational Weight Gain in the Second Trimester of Pregnancy			Continuity between a, b and c
		a: below IOM Recommendations	b: Normal IOM Recommendations	c: above IOM Recommendations	
*n* (%)		757 (14.4)	1522 (29.1)	2959 (56.5)	/
Mean age of mothers (SD) in years		29.6 * (4.2)	30.1 (4)	30.2 (4.4)	/
Mean gestational age at delivery (SD) in months		39.5 (1.4)	39.5 (1.4)	39.4 (1.5)	/
Mothers with no diploma in %		5.3 **	2.1	4.3**	/
Familial annual income <15,500 Canadian dollars in %		3.4	2.3	3.2	/
Maternal outcomes	Gestational diabetes mellitus in %	8.5	6	7.3	0.01
	RR (CI 95%), *p*	1.63 (1.18–2.25), 0.003	1	1.22 (0.95–1.57), 0.13	
	aRR (CI 95%), *p*	1.70 (1.20–2.41), 0.003	1	1.19 (0.90–1.56), 0.22	
	Hypertensive disorders of pregnancy in %	4.1	3	4.9	0.008
	RR (CI 95%), *p*	1.40 (0.88–2.23), 0.16	1	1.68 (1.19–2.36), 0.003	
	aRR (CI 95%), *p*	1.40 (0.84–2.31), 0.20	1	1.80 (1.25–2.59), 0.002	
	Caesarean delivery in %	20.3	17.3	20.9	0.01
	RR (CI 95%), *p*	1.22 (0.98–1.53), 0.075	1	1.27 (1.08–1.49), 0.004	
	aRR (CI 95%), *p*	1.29 (1.01–1.64), 0.039	1	1.21 (1.01–1.44), 0.036	
Neonatal outcomes	Macrosomia in %	8.6	7.9	12.8	<0.001
	RR (CI 95%), *p*	1.10 (0.80–1.50), 0.57	1	1.71 (1.38–2.12), <0.001	
	aRR (CI 95%), *p*	1.13 (0.80–1.58), 0.49	1	1.71 (1.36–2.16), <0.001	
	Large weight for gestational age in %	7.4	7.6	11.9	<0.001
	RR (CI 95%), *p*	0.98 (0.70–1.36), 0.90	1	1.64 (1.32–2.05), <0.001	
	aRR (CI 95%), *p*	1.01 (0.71–1.44), 0.95	1	1.58 (1.24–2.00), <0.001	
	Small weight for gestational age in %	6.4	6.8	4.4	0.001
	RR (CI 95%), *p*	0.92 (0.65–1.32), 0.66	1	0.63 (0.48–0.82), 0.001	
	aRR (CI 95%), *p*	0.83 (0.55–1.25), 0.38	1	0.61 (0.45–0.82), 0.001	
	Neonatal hypoglycemia	1.9	0.9	1.7	0.06
	RR (CI 95%), *p*	2.03 (0.96–4.28), 0.063	1	1.89 (1.04–3.42), 0.036	
	aRR (CI 95%), *p*	2.14 (0.97–4.74), 0.061	1	1.89 (0.99–3.61), 0.052	

aRR: adjusted relative risk; CI: confidence interval; SD: standard deviation. Adjustment of RR was made for all outcomes on age of mothers, parity, smoking during pregnancy, hypertension before pregnancy, diploma of mothers, familial annual incomes and for all outcomes except gestational diabetes mellitus on diabetes before pregnancy. * *p* < 0.05, ** Different from b (*p* < 0.001).

**Table 3:** Association of maternal and neonatal outcomes with IOM recommendations on gestational weight gain in the third trimester of pregnancy.

* Different from b and c (*p* = 0.01)		Gestational Weight Gain in the Third Trimester of Pregnancy			Continuity between a, b and c
		a: below IOM Recommendation	b: Normal (IOM Recommendation)	c: above IOM Recommendation	
*n* (%)		852 (13.3)	2015 (32.9)	3448 (53.8)	/
Mean age of mothers (SD) in years		30.2 (4.2)	30.3 (4.1)	29.8 * (4.4)	/
Mean gestational age at delivery (SD) in months		39.4 (1.2)	39.5 (1.2)	39.5 (1.3)	/
Mothers with no diploma in %		4.6 **	2.3	4.3 **	/
Familial annual income <15,500 Canadian dollars in %		2.9	2.3	3.6 ^	/
Maternal outcomes	Gestational diabetes mellitus in %	10.1	6.1	7.1	0.001
	RR (CI 95%), *p*	1.72 (1.29–2.29), <0.001	1	1.18 (0.94–1.47), 0.15	
	aRR (CI 95%), *p*	1.69 (1.24–2.29), 0.001	1	1.11 (0.87–1.40), 0.41	
	Hypertensive disorders of pregnancy in %	2.7	2.9	5.8	<0.001
	RR (CI 95%), *p*	0.95 (0.58–1.54), 0.82	1	2.09 (1.56–2.80), <0.001	
	aRR (CI 95%), *p*	0.82 (0.48–1.41), 0.48	1	1.91 (1.40–2.61), <0.001	
	Caesarean delivery in %	18	17.6	21.8	0.002
	RR (CI 95%), *p*	1.03 (0.83–1.26), 0.81	1	1.31 (1.14–1.50), <0.001	
	aRR (CI 95%), *p*	1.11 (0.89–1.39), 0.35	1	1.34 (1.15–1.56), <0.001	
Neonatal outcomes	Macrosomia in %	5.6	7.3	13.8	<0.001
	RR (CI 95%), *p*	0.76 (0.54–1.06), 0.10	1	2.03 (1.68–2.46), <0.001	
	aRR (CI 95%), *p*	0.78 (0.55–1.12), 0.18	1	2.17 (1.77–2.67), <0.001	
	Large weight for gestational age in %	4.9	6.6	13.3	<0.001
	RR (CI 95%), *p*	0.73 (0.51–1.04), 0.08	1	2.16 (1.77–2.63), <0.001	
	aRR (CI 95%), *p*	0.68 (0.46–1.01), 0.05	1	2.26 (1.83–2.80), <0.001	
	Small weight for gestational age in %	9.5	6.5	4.1	<0.001
	RR (CI 95%), *p*	1.52 (1.14–2.02), 0.004	1	0.62 (0.48–0.79), <0.001	
	aRR (CI 95%), *p*	1.48 (1.07–2.06), 0.019	1	0.58 (0.45–0.77), <0.001	
	Neonatal hypoglycemia	1.2	1.1	2	0.009
	RR (CI 95%), *p*	1.12 (0.53–2.38), 0.76	1	1.96 (1.21–3.18), 0.006	
	aRR (CI 95%), *p*	1.32 (0.61–2.85), 0.49	1	1.89 (1.12–3.18), 0.017	

aRR: adjusted relative risk; CI: confidence interval; SD: standard deviation. Adjustment of RR was made for all outcomes on age of mothers, parity, smoking during pregnancy, hypertension before pregnancy, diploma of mothers, familial annual incomes and for all outcomes except gestational diabetes mellitus on diabetes before pregnancy. * Different from a and b (*p* < 0.001); ** Different from b (*p* < 0.001); ^ Different from b (*p* < 0.05).

**Table:** **(A)**

		T2H (*n* = 2886)		*p*
		T2H ≥ T3N or T3L	T2H ≥ T3H	
*n* (%)		616 (21.3)	2270 (78.7)	/
Mean age of mothers (SD) in years		30.9 (4.2)	30 (4.5)	<0.001
Mean gestational age at delivery (SD) in months		39.6 (1.2)	39.5 (1.3)	0.11
Maternal outcomes	Gestational diabetes mellitus in %	8.8	6.9	0.12
	Hypertensive disorders of pregnancy in %	2.8	5.3	0.008
	Caesarean delivery in %	16.9	22	0.006
Neonatal outcomes	Macrosomia in %	8.3	14.2	<0.001
	Large weight for gestational age in %	7	13.2	<0.001
	Small weight for gestational age in %	6.2	3.8	0.01
	Neonatal hypoglycemia in %	2.1	1.7	0.47

SD: standard deviation; T2H: weight gain above IOM recommendations in second trimester; T3N: weight gain following IOM recommendations in third trimester; T3H: weight gain above IOM recommendations in third trimester; T3L: weight gain below IOM recommendations in third trimester.

**Table:** **(B)**

		T2H (*n* = 1488)		*p*
		T2H ≥ T3N or T3L	T2H ≥ T3H	
*n* (%)		1131 (76)	357 (24)	/
Mean age of mothers (SD) in years		30.4 (3.9)	29.2 (3.9)	<0.001
Mean gestational age at delivery (SD) in months		39.5 (1.2)	39.5 (1.2)	0.97
Maternal outcomes	Gestational diabetes mellitus in %	5.5	7	0.29
	Hypertensive disorders of pregnancy in %	2.2	5.3	0.002
	Caesarean delivery in %	16.8	18.2	0.54
Neonatal outcomes	Macrosomia in %	6.8	11.8	0.003
	Large weight for gestational age in %	6.2	12.4	<0.001
	Small weight for gestational age in %	7.4	4.5	0.06
	Neonatal hypoglycemia in %	0.6	2	0.02

SD: standard deviation; T2N: weight gain following IOM recommendations in second trimester; T3N: weight gain following IOM recommendations in third trimester; T3H: weight gain above IOM recommendations in third trimester; T3L: weight gain below IOM recommendations in third trimester.

**Table 5:** Characteristics of weight gain trajectories during pregnancy identified by group-based multi-trajectory modelling.

		Weight Gain Trajectories			*p*
		Profile A	Profile B	Profile C	
*n* (%)		3819 (58.3)	2124 (32.4)	608 (9.3)	/
Mean age of mothers (SD) in years		29.9 (4.2)	30.1 (4.4)	30.1 (4.3)	0.17
Mean gestational age at delivery (SD) in months		39.4 (1.4)	39.5 (1.5)	39.3 (1.6)	0.001, A vs. B and C, B vs. C
Mean of pre-pregnancy BMI (SD) in kg·m^−2^		21.2 (2.2)	26.3 (3.1)	35.6 (5)	<0.001, A vs. B and C, B vs. C
Mothers with no diploma in %		3.2	2.7	3.7	NS
Familial annual income <15,500 Canadian dollars in %		3.5	3.9	5.2	NS
Pre-pregnancy BMI (in kg·m^−2^) intervals in %	<18.5 (Underweight)	9	0	0	<0.001
	18.5–24.9 (Normal)	85.6	36.5	0.2	
	25–29.9 (Overweight)	5.4	50.9	10.5	
	>30 (Obese)	0	12.6	89.3	
Median of gestational weight gain (min, max, IQR) in kg	First trimester	2 (−9.5, 21, 1–3.7)	2.7 (−9, 20.6, 1–4.9)	1.7 (−10.8, 28.6, 0–4)	0.001, B versus A and C
	Second trimester	8.8 (−5, 28, 6.8–11)	9.5 (−11, 28, 7–12.8)	7 (−9.6, 38.7, 3.5–11)	0.001, A versus B and C, B vs. C
	Third trimester	14 (−0.5, 40.3, 11.6–17)	15.9 (−11.1, 44, 12–19.5)	13 (−9.6, 45.7, 7.7–17.7)	0.001, A versus B and C, B vs. C

BMI: body mass index; IQR: interquartile range; SD: standard deviation; NS: Not significant.

**Table 6:** Association of maternal and neonatal outcomes with weight gain trajectories during pregnancy identified by group-based multi-trajectory modelling.

		Weight Gain Trajectories			Continuity between A, B and C
		Profile A	Profile B	Profile C	
Maternal outcomes	Gestational diabetes mellitus in %	5.3	8.1	16.5	<0.001
	RR (CI 95%), *p*	1	1.58 (1.29–1.95), <0.001	3.52 (2.73–4.56), <0.001	
	aRR (CI 95%), *p*	1	1.44 (1.14–1.80), 0.002	3.19 (2.40–4.23), <0.001	
	Hypertensive disorders of pregnancy in %	2.9	5.3	11.4	<0.001
	RR (CI 95%), *p*	1	1.88 (1.43–2.46), <0.001	4.32 (3.15–5.91), <0.001	
	aRR (CI 95%), *p*	1	1.87 (1.41–2.49), <0.001	3.66 (2.56–5.24), <0.001	
	Caesarean in %	18.5	20.6	28.5	<0.001
	RR (CI 95%), *p*	1	1.15 (1–1.31), 0.043	1.76 (1.45–2.13), <0.001	
	aRR (CI 95%), *p*	1	1.12 (0.97–1.30), 0.13	1.74 (1.40–2.16), <0.001	
Neonatal outcomes	Macrosomia in %	6.6	14.3	22	<0.001
	RR (CI 95%), *p*	1	2.36 (1.98–2.81), <0.001	3.96 (3.14–4.99), <0.001	
	aRR (CI 95%), *p*	1	2.24 (1.85–2.71), <0.001	3.86 (3.00–4.97), <0.001	
	Large weight for gestational age in %	5.9	13.8	22.2	<0.001
	RR (CI 95%), *p*	1	2.54 (2.12–3.05), <0.001	4.54 (3.59–5.74), <0.001	
	aRR (CI 95%), *p*	1	2.42 (1.99–2.94), <0.001	4.29 (3.31–5.55), <0.001	
	Small weight for gestational age in %	6.9	4	3.2	<0.001
	RR (CI 95%), *p*	1	0.56 (0.43–0.71), <0.001	0.44 (0.27–0.70), 0.001	
	aRR (CI 95%), *p*	1	0.58 (0.44–0.77), <0.001	0.40 (0.23–0.69), 0.001	
	Neonatal hypoglycemia	1.4	1.5	2.6	0.11
	RR (CI 95%), *p*	1	1.06 (0.69–1.66), 0.78	1.88 (1.07–3.31), 0.028	
	aRR (CI 95%), *p*	1	1.13 (0.71–1.81), 0.61	1.88 (1.01–3.49), 0.045	

aRR: adjusted relative risk; CI: confidence interval. Adjustment of RR was made for all outcomes on age of mothers, parity, smoking during pregnancy, hypertension before pregnancy, diploma of mothers, familial annual incomes and for all outcomes except gestational diabetes mellitus on diabetes before pregnancy.
